# From innovative applications of the effectiveness-implementation hybrid trial design to the dissemination, implementation, effectiveness, sustainment, economics, and level-of-scaling hybrid trial design

**DOI:** 10.3389/frhs.2022.1007750

**Published:** 2022-12-06

**Authors:** Bryan R. Garner

**Affiliations:** Department of Internal Medicine, The Ohio State University, Columbus, OH, United States

**Keywords:** hybrid effectiveness-implementation trials, mixed-methods evaluation, knowledge translation, Type 1 hybrid trial, Type 2 hybrid trial, Type 3 hybrid trial, DIeSEL hybrid trial

## Abstract

To address the enduring gap between research and practice, there is a need to improve the speed and efficiency of research across the translational research spectrum. In 2012, the effectiveness-implementation hybrid trial design (HTD) was codified as a design with the potential to improve the speed and efficiency of translation, especially as part of T2 (clinical research) translational research. Building on this and other recent efforts to stimulate greater use of this novel HTD, the current article highlights an innovative application of each effectiveness-implementation HTD type. The completed application of the Type 1 effectiveness-implementation HTD tested the effectiveness of a clinical intervention for reducing HIV viral load and retaining people with HIV in care, as well as conducted a longitudinal mixed-methods examination to test for significant changes over time in three key measures of context, and economic evaluation of the clinical intervention. The completed application of the Type 2 effectiveness-implementation HTD used a dual-randomized design to simultaneously test the effectiveness of a clinical intervention for addressing substance use disorder among people with HIV and effectiveness of a blended strategy called the Implementation and Sustainment Facilitation (ISF) Strategy. This Type 2 hybrid trial was also innovative due to its focus on both sustainment and economic outcomes. The innovative Type 3 application (funded in 2008 and completed in 2012) tested the effectiveness and cost-effectiveness of using pay-for-performance to improve both implementation outcomes and client outcomes. This article also codifies a HTD called the Dissemination, Implementation, effectiveness, Sustainment, Economics, and Level-of-scaling (DIeSEL) HTD.

## Introduction

Guided by their “experience over many years in writing, reviewing, and conducting research projects across the efficacy-effectiveness-implementation spectrum”. Curran et al. (1) codified the effectiveness-implementation hybrid trial design (HTD) as a design with the “potential to speed and improve translation.” Since it was published a decade ago, the Curran et al. (1) article has been cited over 2,000 times, suggesting the codification of the effectiveness-implementation HTD has had a significant impact on the field and does have the potential to speed and improve translation. More recently, Landes et al. (2) published an introduction to the effectiveness-implementation HTD and provided examples of its three types (i.e., Type 1, Type 2, and Type 3). Building on this prior work, the current article highlights several innovative applications of each effectiveness-implementation HTD type, as well as codifies a novel Dissemination, Implementation, effectiveness, Sustainment, Economic, and Level-of-scaling (DIeSEL) HTD (3), which is an advancement of the effectiveness-implementation HTD that received a “Best of” distinction at the 2022 Colorado Pragmatic Research in Health Conference.

## Innovative applications of the effectiveness-implementation hybrid trial design

One innovative application of the Type 3 effectiveness-implementation HTD is provided by the Reinforcing Therapist Performance (RTP) Experiment (4). Funded by the National Institute on Alcohol Abuse and Alcoholism in 2008, 4 years before Curran et al. (1) introduced their HTD, this 29-site cluster-randomized trial (i.e., Type 3 effectiveness-implementation HTD) experimentally tested a financing strategy called pay-for-performance (P4P) as a strategy to improve both implementation outcomes and client outcomes. As detailed by Garner et al. (5) compared to the control implementation-as-usual (IAU) strategy (i.e., training, feedback, and on-going consultation regarding the clinical intervention for addressing adolescent substance use), the experimental IAU+P4P strategy was found to have a significant direct impact on improving the staff-level implementation outcomes and a significant indirect impact on improving the client-level outcome. Beyond being conceptualized and completed prior to the formal codification in 2012, this application of the Type 3 effectiveness-implementation HTD is further innovative in that it included an economic aim to test the cost-effectiveness of the P4P strategy (4). As detailed by Garner et al. (6) although the P4P strategy led to a significantly higher average total cost, the average increase of 5% resulted in a 116% increase in the average number of months staff demonstrated competence (i.e., fidelity) regarding implementation of the clinical intervention with clients, as well as a 325% increase in the average number of clients who received the targeted number (i.e., dosage) of treatment sessions and the number of days of abstinence per patient in treatment. Supporting the cost-effectiveness of the P4P strategy further, the cost per Quality Adjusted Life Year (QALY) was only $861.

With regards to the Type 2 effective-implementation HTD, a highly innovative application is provided by Substance Abuse Treatment to HIV Care (SAT2HIV) Project, a 39-site dual-randomized Type 2 effectiveness-implementation hybrid trial funded in 2014 by the National Institute on Drug Abuse (7, 8). As detailed by Garner et al. (7) the implementation aim focused on testing the Implementation and Sustainment Facilitation (ISF) Strategy as an adjunct to the strategy empirically supported by Miller et al. (9) and used by Addiction Technology Transfer Centers (ATTCs) for training staff in motivational interviewing. As detailed by Garner et al. (8) the effectiveness aim focused on testing a single-session 15–30 min motivational interviewing-based brief intervention (MIBI) for addressing substance use disorders among people with HIV as an adjunct to usual care within HIV service organizations. Made possible *via* the innovative dual-randomized design, Garner et al. (10) found a significant cross-level interaction where the ISF Strategy had a significant impact on improving the consistency and quality of MIBI implementation by the trained staff (i.e., implementation effectiveness) and the effectiveness of the MIBI for reducing days of substance use (i.e., intervention effectiveness). Thus, more use of the innovative dual-randomized Type 2 effectiveness-implementation HTD is warranted and has the potential to help better understand not only what strategies improve implementation outcomes and client outcomes, but what strategies might be able to minimize the decreased intervention effectiveness (i.e., voltage drop) often found when interventions are implemented without the resources and supports included as part of the efficacy research. The SAT2HIV Project's application of the Type 2 effectiveness-implementation HTD was further innovative due to its expanded focus on sustainment and economics (7). The ISF Strategy was not found to have a significant impact on sustainment (10), but did have some evidence to support its cost-effectiveness to improve implementation quality. More specifically, as detailed by Hinde et al. (11) the ISF Strategy's incremental cost per-staff of $2,457 divided by the incremental difference in implementation quality per staff of 61.45 resulted in an incremental cost-effectiveness ratio of $40, which according to sensitivity analyses has a 71% probability of being cost-effective.

Regarding the Type 1 effectiveness-implementation HTD, an innovative application is provided by the Positive Health Check (PHC) Project (12). Funded by the Centers for Disease Control and Prevention (CDC) in 2014, the primary aim of this four-site trial was to test the effectiveness of the PHC intervention, which is a computer-delivered intervention developed for reducing HIV viral load and retaining people with HIV in care, and the secondary aim was to longitudinally assess inner setting context measures (i.e., innovation-values fit, organizational readiness for implementing change, and implementation climate) that were hypothesized to change over the course of the effectiveness trial. Beyond being one of the first to formally use the Type 1 effectiveness-implementation HTD, the PHC Project's application is innovative in at least two additional ways. First, it is innovative because it expanded the Type 1 effectiveness-implementation HTD to include an economic aim focused on cost and cost-effectiveness (12). Second, it is innovative because it overcame the “small n” problem (i.e., only having four sites) noted by Proctor et al. (13) by longitudinally assessing these measures at eight timepoints, which enabled sufficiently powered statistical tests of the extent to which there were statistically significant linear or curvilinear changes over time in the three key contextual constructs posited to be important according to the theory of implementation effectiveness (14–16). More specifically, both quantitative surveys and qualitative interviews were conducted at baseline and then every 3 months over a 23-month period to collect the eight data time points used to test for statistically significant changes over time in an innovation-values fit measure developed for the PHC intervention (12), a measure of organizational readiness for implementation change developed by Shea et al. (17) and a measure of implementation climate developed by Jacob et al. (18). As recently reported by Garner et al. (19) there were not a significant changes over time found for innovation-values fit or organizational readiness for implementing change, but there was significant change over time for implementation climate.

## The DIeSEL hybrid trial design

**Figure 1:**
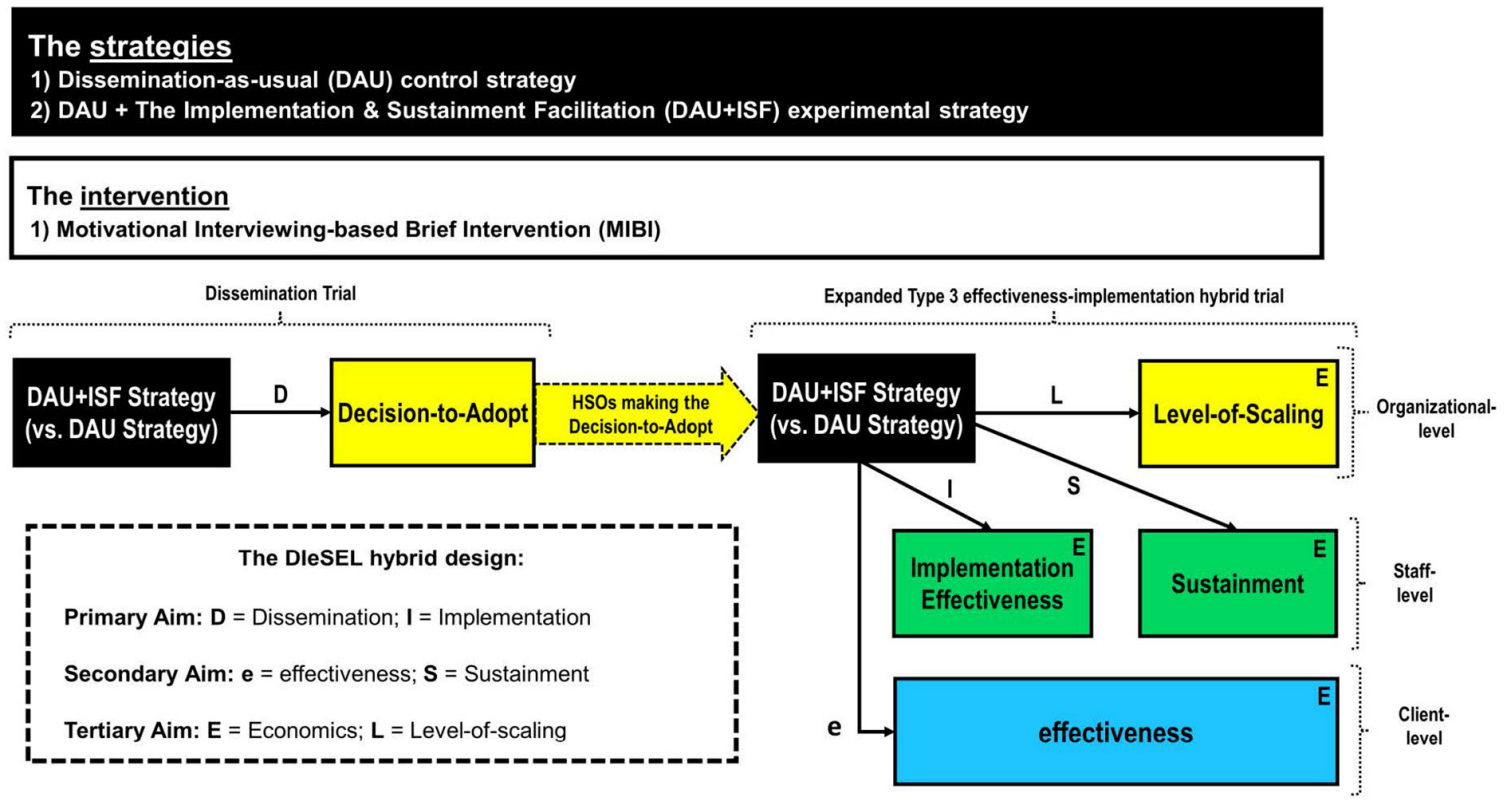
Visual representation of the study design and aims.

Based on my experience with the effectiveness-implementation HTD, which beyond the three innovative applications highlighted in the prior section includes two Type 3 effectiveness-implementation hybrid trials (20, 21), and a Type 1 effectiveness-implementation hybrid trial (22), I would attest that the first two HTD types (Type 1, Type 2) can help improve the speed and efficiency of translation, especially as part of translational research focused on the T2 (clinical research) stage of the translational research spectrum (23). Additionally, I would attest that the third type (Type 3) can help improve the speed and efficiency of translation, especially as part of translational research focused on the T3 (clinical implementation) stage (23). In response to Curran et al. (1) “hope to stimulate further thinking and to encourage new design combinations” and the current call for proposed advancements to the effectiveness-implementation HTD, this article also codifies the DIeSEL HTD as a novel HTD with potential to further improve the speed and efficiency of translation, especially during T3 (clinical implementation), but perhaps also as part of T4 (public health) translational research. The DIeSEL HTD was codified for use as part of the NIDA-funded Substance Treatment Strategies for HIV (STS4HIV) Project, which since being funded in 2018 has completed three stakeholder-engaged real-time Delphi (SE-RTD) surveys to empirically identify the substance use disorders with the most negative population-level impact (24), the best fitting evidence-based interventions for integration into HIV service settings (25), and the best fitting strategies for the AIDS Education and Training Centers (AETC) purveyor network to use in helping improve the integration of evidence-based substance use disorder interventions within HIV service settings (26). Organized by aim, below is an overview of the this HTD, which as Figure 1 helps visualize, is essentially a dissemination trial combined with a Type 3 effectiveness-implementation hybrid trial expanded to also combine elements of sustainment research, economic research, and scaling research.

### Primary aim

The primary aim is to experimentally test the impact of the ISF Strategy on decision-to-adopt (path D of Figure 1), the primary dissemination outcome, and implementation effectiveness (path I of Figure 1), the primary implementation outcome. Consistent with the National Institutes of Health (NIH) definition of dissemination research (i.e., the scientific study of targeted distribution of information and intervention materials to a specific public health or clinical practice audience, an initial step of a DIeSEL HTD is establishment of an engaged community and organizational shareholders (ECOS; i.e., the specific health or clinical practice audience), which provides the denominator for the dissemination experiment. Notably, shareholders is used in place of stakeholders given that some have suggested the term stakeholder be banished (27). For the STS4HIV Project, the targeted ECOS is HIV service organizations (HSOs) from across the United States. To include as many HSOs as possible, establishment of the STS4HIV Project's ECOS will be done in partnership with AETCs and the AETC National Coordinating Resource Center. Thus, all known HSOs will be emailed an invitation about the project and invited to be part of its ECOS. HSOs agreeing to be part of the ECOS will be asked to complete a brief background form that is used to collect organizational-level information about the HSO, including their perceived need for integrating a MIBI within their HSO. Thus, the background information will be useful for providing descriptive statistics about the HSO's included as part of the ECOS, ensuring randomization is balanced, and detection and understanding of disparities regarding any of the project's dissemination, implementation, effectiveness, sustainment, or level-of-scaling outcomes.

Once the project's ECOS has been established, each of the included HSOs will be randomized to either the dissemination-as-usual (DAU) condition or the dissemination-as-usual + ISF Strategy (DAU+ISF) condition. The DAU strategy includes emailing the HSO's designated primary contact the project's recommendation and resources document, which based on the project's prior findings will recommend and provides free resources (e.g., manual, online training modules) for the MIBI that was found to be effective as part of the previously described dual-randomized Type 2 effectiveness-implementation hybrid trial (10). In addition to the DAU strategy, HSOs randomized to the DAU+ISF strategy will receive an email and/or call from one of the project's trained facilitators to communicate that the HSO may receive facilitation at no charge to their HSO. During the project's 2-month exploration phase, each HSO may receive up to 3 h of facilitation. During the subsequent 10-month preparation/implementation phase, HSOs may receive up to 20 additional hours of facilitation, with carry-over of any unused time from the exploration phase being allowed. As part of the 2-month exploration phase, the facilitator will use the strategy's guiding principles (i.e., engage, focus, evoke, plan) and menu of exercises (e.g., decisional balance, past implementation effort) to assist the HSO in deciding whether or not to make the decision-to-adopt the MIBI. Across both conditions, HSOs responding no will be asked to provide the reason(s) and HSOs responding yes will be asked to complete a brief online organizational survey to collect additional information about their HSO, including the primary reason(s) for making the formal decision-to-adopt the MIBI, and the staff they would like to have access to the project's MIBI training resources (i.e., the HSO's targeted/designated users).

Upon completion of the project's 2-month exploration phase, for the HSOs that made the formal decision to adopt the MIBI, the HSO's designated staff will be emailed the project's MIBI resources link, which includes the MIBI manual, an introductory online training module, and an intermediate online training module. Continuing education credits will be made available to staff for completion of each online training. Again, HSO's and their designated staff randomized to the DAU+ISF condition will receive free assistance (i.e., facilitation) by one of the project's trained facilitators. Consistent with the frequency and duration provided as part of the SAT2HIV Project, the default will be to offer HSO's monthly 30–60 min virtual ISF meetings. However, to be as organized-centered as possible, HSO's will be informed that they may utilize their allotted hours of facilitation support however works best for them and that can be accommodated by the project's team. After one of the HSO's staff has successfully completed the two online training modules, the trained staff will be provided access to an HSO-specific Lyssn platform account. The Lyssn platform, which was also used as part of the SAT2HIV-II Project (28), enables secure uploading of digital recordings of MIBI sessions and uses artificial intelligence to rate the quality/fidelity of the MIBI session. Consistent with the SAT2HIV Project (10) and the SAT2HIV-II Project, Garner et al. (21) staff-level implementation effectiveness (i.e., the consistency and quality of implementation by targeted users), which is the primary implementation outcome, will be computed for each of the HSO's designated staff by summing together their standardized sum number of MIBI sessions implemented with clients (i.e., consistency) and their standardized sum fidelity score (i.e., quality).

### Secondary aim

The secondary aim is to test the impact of the ISF Strategy on client-level change in days of primary substance use (path e of Figure 1), the primary effectiveness outcome, as well as on staff-level sustainment status (path S of Figure 1), which is the primary sustainment outcome. Clients who receive a MIBI session from one of the HSO's trained MIBI staff will be invited to participate in the project and complete two brief online surveys. The first is to be completed within 24 h of the MIBI session and the second approximately 4-weeks later. The client-level change in days of primary substance use score, will be computed by subtracting the client's number of days of primary substance use at baseline from their number of days of primary substance use at follow-up. Negative scores therefore indicate a reduction in client's days of primary substance use and positive scores indicate an increase.

For each participating HSO staff, their employment status (1 = employed; 0 = not employed) and training status (1 = training completed; 0 = training not completed) at the end of the project's implementation phase will be documented. This approach is guided by prior research focused on training and retaining staff to competently deliver an EBP (29). Employment status will be based on the HSO's administrative records, while training status will be based on the project's training records. Notably, sustainment research by Hunter et al. (30) found that an organization's number of trained staff sustained at the end of the implementation phase was one of the most significant indicators of longer-term sustainment.

### Tertiary aim

The tertiary aim is to evaluate the ISF Strategy's cost-effectiveness (see each E in Figure 1), the primary economic outcome, as well as to test the impact of the ISF Strategy on organizational-level scale-up of the MIBI (see L in Figure 1), which is the primary level-of-scaling outcome. Information on the quantity of resources used (e.g., labor) will be collected using data from project records, HSO administrative records, the ISF Strategy's implementation tracking system, time-stamped recordings of MIBI sessions, and staff surveys. The cost-effectiveness of the ISF Strategy will be assessed using incremental cost-effectiveness ratios (ICERs) and cost-effectiveness acceptability curves (CEACs). These will be calculated from the payer perspective, which for the AETC network is the Health Resources and Services Administration. Client outcomes will be converted into quality-adjusted life years using disability weights from the literature. The most cost-effective strategy will be the one with the largest ICER that falls below a threshold valued by decision makers on an additional unit of effect for the respective outcome.

**Figure 2:**
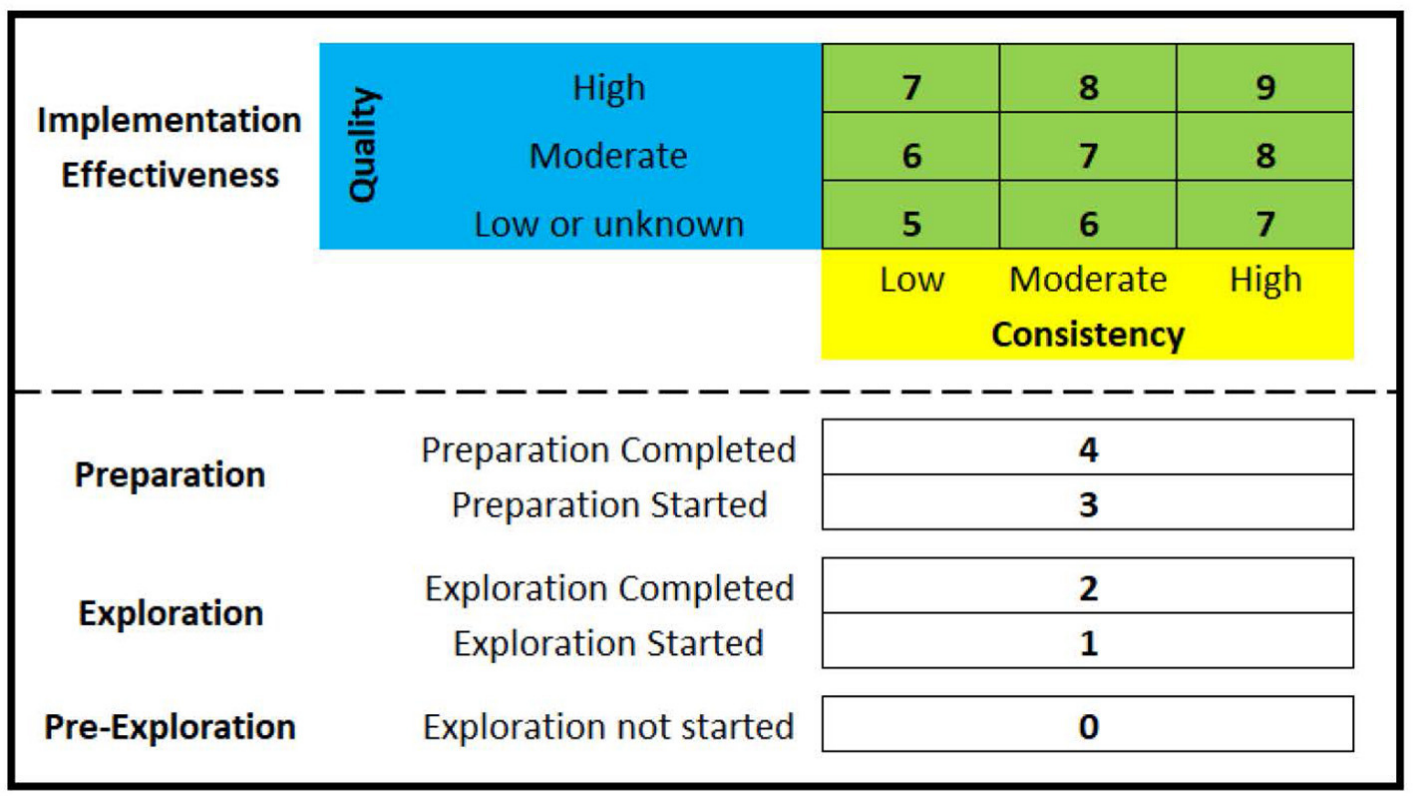
The Exploration, Preparation, Implementation Effectiveness (EPIE) measure.

As highlighted as part of the project's Implementation Research Logic Model (25), the planned approach for assessing HSO's scale-up of evidence-based interventions for substance use disorders was to focus on overall change between baseline and the end of the implementation phase using a novel 5-point EPIS measure that includes pre-exploration as a phase (0 = pre-exploration, 1 = exploration, 2 = preparation, 3 = implementation, 4 = sustainment). However, this measure is being replaced with the Exploration, Preparation, Implementation Effectiveness (EPIE) measure, which as illustrated in Figure 2 is a novel 10-point measure that retains capture of a pre-exploration phase, makes the distinction between “started” and “completed” for both the exploration phase and the preparation phase, as well as makes a distinction between five levels of implementation effectiveness. The EPIE measure is therefore a pragmatic measure that enables better differentiation between HSOs not scaling to the implementation phase, as well as better differentiation between HSOs scaling to the implementation phase but that differ in terms of implementation effectiveness (i.e., the consistency and quality of implementation). Once an HSO submits a formal decision-to-adopt the MIBI, the HSO will be documented as a 2 on the EPIE measure (i.e., exploration phase completed). To test the impact of the ISF Strategy on the HSO's level of MIBI scale-up, upon completion of the project's preparation/implementation phase each of the HSOs that made the formal decision-to-adopt the MIBI will be asked to participate in a qualitative interview focused on understanding how the HSO's status on the EPIE measure has changed (if at all) since the end of the exploration phase. Thus, the EPIE measure will enable a novel way of assessing levels-of-scaling that is consistent with both the EPIS framework (31) and the theory of implementation effectiveness (14–16).

## Conclusions

To help improve the speed and efficiency of translation, researchers are recommended to use the effectiveness-implementation HTD as part of T2 (clinical research) translational research and the DIeSEL HTD as part of T3 (clinical implementation) translational research.

## Author contributions

The author confirms being the sole contributor of this work and has approved it for publication.

## Funding

This work was funded by the National Institute on Drug Abuse (R01-DA044051).

## Conflict of interest

The author declares that the research was conducted in the absence of any commercial or financial relationships that could be construed as a potential conflict of interest.

## Publisher's note

All claims expressed in this article are solely those of the authors and do not necessarily represent those of their affiliated organizations, or those of the publisher, the editors and the reviewers. Any product that may be evaluated in this article, or claim that may be made by its manufacturer, is not guaranteed or endorsed by the publisher.

## Author disclaimer

The content is solely the responsibility of the author and does not necessarily represent the official views of the National Institute on Drug Abuse.
